# Perspectives on High-Throughput Ligand/Protein Docking With Martini MD Simulations

**DOI:** 10.3389/fmolb.2021.657222

**Published:** 2021-03-29

**Authors:** Paulo C. T. Souza, Vittorio Limongelli, Sangwook Wu, Siewert J. Marrink, Luca Monticelli

**Affiliations:** ^1^Groningen Biomolecular Sciences and Biotechnology Institute and Zernike Institute for Advanced Materials, University of Groningen, Groningen, Netherlands; ^2^PharmCADD, Busan, South Korea; ^3^Molecular Microbiology and Structural Biochemistry (MMSB, UMR 5086), CNRS, University of Lyon, Lyon, France; ^4^Faculty of Biomedical Sciences, Institute of Computational Science, Università della Svizzera Italiana (USI), Lugano, Switzerland; ^5^Department of Pharmacy, University of Naples “Federico II”, Naples, Italy; ^6^Department of Physics, Pukyong National University, Busan, South Korea

**Keywords:** molecular dynamics, coarse-grain, ligand-protein, protein-protein interaction, Martini, dynamic docking, high-throughput screening, drug design

## Abstract

Molecular docking is central to rational drug design. Current docking techniques suffer, however, from limitations in protein flexibility and solvation models and by the use of simplified scoring functions. All-atom molecular dynamics simulations, on the other hand, feature a realistic representation of protein flexibility and solvent, but require knowledge of the binding site. Recently we showed that coarse-grained molecular dynamics simulations, based on the most recent version of the Martini force field, can be used to predict protein/ligand binding sites and pathways, without requiring any *a priori* information, and offer a level of accuracy approaching all-atom simulations. Given the excellent computational efficiency of Martini, this opens the way to high-throughput drug screening based on dynamic docking pipelines. In this opinion article, we sketch the roadmap to achieve this goal.

## Introduction

Structure-based drug design has been extensively used by pharmaceutical companies and academic research groups to reduce the cost and time necessary for the discovery of new drugs. The approach relies on the knowledge of the atomistic structure of the biological target, obtained by experiments (e.g., X-ray crystallography, NMR spectroscopy, cryo-electron microscopy) or modeling (e.g., based on homology). Standard pipelines often start with *in silico* docking experiments, used for virtual screening of thousands of compounds or molecular fragments (Sliwoski et al., 2014; Leelananda and Lindert, 2016; Duarte et al., 2019). After a significant reduction of the chemical space, a selected group of molecules can be optimized by all-atom (AA) molecular dynamics (MD) based simulations (Jorgensen and Thomas, 2008; Jorgensen, 2009; Vivo et al., 2016; Limongelli, 2020). AA MD simulations can be used not only to improve the prediction of the binding pose and affinity, but also to get insight into (un)binding rates and pathways (Dror et al., 2011; Shan et al., 2011; Limongelli et al., 2013; Tiwary et al., 2015; Copeland, 2016; Bruce et al., 2018). As a third step, further selection can be performed considering predictions of absorption, distribution, metabolism, excretion and toxicity (ADMET) (Van De Waterbeemd and Gifford, 2003; Cheng et al., 2013). The obtained lead compounds need to be validated by *in vitro* assays and structurally improved – lead optimization – to achieve drug candidates, which are tested in animal models and eventually enter clinical trials before final approval. Despite the rapid advances in computer-aided drug discovery methods, the limitations of such approaches are still major. Docking assays remain to date the first option in the drug discovery pipeline thanks to their capability of “virtually” testing thousands of molecules in a short time. However, docking accuracy is poor due to limitations in simplified energy (“scoring”) functions, sampling ligand and protein flexibility (Grinter and Zou, 2014), and representation of the environment – crucial in hydrated binding pockets and in transmembrane proteins, that represent a large fraction of the pharmaceutically relevant protein targets. AA MD simulations can tackle these limitations, but they are still computationally prohibitively expensive (Durrant and McCammon, 2011; Liu et al., 2018; Miller et al., 2020), due to relatively long time scales of conformational dynamics in proteins. Moreover, predictions of dissociation pathways and rates are extremely challenging, and require high performance computing and enhanced sampling techniques (Limongelli et al., 2013; Casasnovas et al., 2017; Brotzakis et al., 2019; Schuetz et al., 2019). Peptide and protein design for biopharmaceutical applications have similar pitfalls, with the current approaches reasonably successful in predicting protein structures (Hutson, 2019; Callaway, 2020) and rigid-body protein-protein interactions (Siebenmorgen and Zacharias, 2020) but with limitations in the design of conformational changes (Feldmeier and Höcker, 2013; Yang and Lai, 2017; Perkel, 2019; D’Annessa et al., 2020).

Coarse-grained (CG) modeling is a computationally cheaper alternative to high-resolution atomistic approaches (Ingólfsson et al., 2014; Kmiecik et al., 2016), as it reduces the computational cost by grouping atoms into effective interaction sites. Numerous CG models have been developed during the past two decades, with different levels of coarsening and different mathematical representations. CG models have been successfully applied to study a large range of processes in biology (Yen et al., 2018; Bruininks et al., 2020; Lucendo et al., 2020) and materials science (Casalini et al., 2019; Alessandri et al., 2020; Li et al., 2020; Vazquez-Salazar et al., 2020). Applications such as structure-based drug design are particularly challenging for CG modeling because of the severe requirements: (1) high chemical specificity (i.e., allowing to distinguish most chemical groups); (2) capability to represent all possible components of the system (proteins, cofactors, nucleic acids, drug candidates, waters, lipids, etc.) in a coherent way; (3) realistic representation of conformational flexibility of each molecule in the system; and (4) accurate thermodynamics and kinetics of binding. Currently, none of the CG force fields available fulfills all the requirements above, but the Martini CG force field fulfills at least some (Marrink et al., 2007; Marrink and Tieleman, 2013), as it allows modeling all main biomolecules (Monticelli et al., 2008; López et al., 2009; de Jong et al., 2013, 2015; Uusitalo et al., 2015, 2017; Wassenaar et al., 2015) with relatively high chemical specificity, and proteins may still retain reasonable conformational flexibility (Periole et al., 2009; Melo et al., 2017; Poma et al., 2017). As in AA MD simulations, most of the details of the environment can be included in Martini CG simulations, for instance an explicit solvent model or a complex bilayer composition (Ingolfsson et al., 2015; Marrink et al., 2019).

Although Martini-based CG MD simulations have been used to study a wide range of biomolecular processes, examples of protein–ligand binding are still scarce (Negami et al., 2014, 2020; Delort et al., 2017; Ferré et al., 2019; Jiang and Zhang, 2019; Dandekar and Mondal, 2020). Studies of protein-protein interactions are more common, although usually restricted to membrane environments (Baaden and Marrink, 2013; Castillo et al., 2013; Lelimousin et al., 2016; Sun et al., 2020). In some cases, binding of lipids to sites deeply buried inside the protein can be obtained by brute force Martini MD (Arnarez et al., 2013; Van Eerden et al., 2017; Corradi et al., 2019). Overall, some limiting factors hampered the use of Martini in small-molecule and protein design: (1) chemical specificity to reproduce the broad chemical space of drugs; (2) the thermodynamics of ligand-protein and protein-protein interactions are generally overestimated (Stark et al., 2013; Javanainen et al., 2017; Alessandri et al., 2019); and (3) introduction of conformational flexibility in proteins requires case-by-case optimization (Negami et al., 2020; Ahalawat and Mondal, 2021). A new version of the Martini force field, named Martini 3 (Souza et al., 2021), partly solves these issues: it can represent a broader variety of chemical compounds, and it features improved molecular packing and optimized molecular interactions (along with specific interactions mimicking H-bonding and electronic polarizability). Recently, Martini 3 was successfully applied to a range of protein-ligand system examples, from the well-characterized T4 lysozyme to members of the GPCR family and nuclear receptors to a variety of enzymes (Souza et al., 2020). In addition, combination of Martini 3 and Gō-like potentials can substantially improve the modeling of protein flexibility (Poma et al., 2017; Souza et al., 2019). Combined, these new features open the possibility of computer-aided drug design based on CG models.

In this perspective, we sketch a possible roadmap for a drug design pipeline using Martini, where no *a priori* information about the target pocket is necessary. Competition between ligands for different pockets and environments can be included in the screening. Protein flexibility can be incorporated to a certain degree, allowing the possible discovery of cryptic (hidden) pockets (Kuzmanic et al., 2020). Ligand (un)binding pathways are accessible via enhanced sampling techniques (Raniolo and Limongelli, 2020), and enable for the first time the possibility of a “dynamic” drug screening based not only on ligand binding modes, but also on kinetically relevant states – that is considering binding affinity and dissociation rates (i.e., drug residence time). The next sections detail the key steps of this pipeline.

## Ligand Databases: Coarse-Graining the Ligands

The very first step to develop a Martini drug design pipeline is to create curated and validated databases containing hundreds to thousands of small-molecule models. This CG database needs to include molecular moieties usually found in drugs, such as halogens, heterocycles, and sulfamides. Alternatively, the databases of low-molecular-weight molecules (∼150 Da) can also be created for fragment-based drug discovery campaigns (Rognan, 2012). Parameters for molecules/fragments of pharmaceutical interest need to be validated by comparison between CG, AA and, if available, experimental data for a subset of relevant target systems. Once validated, all the models will be made available via the open-access Martini Database (MAD) web server^1^. The initial CG databases are also the foundation to develop and calibrate automatic tools to generate parameters for new CG models. Such automatic tools should perform AA to CG mapping [as performed by auto-martini (Bereau and Kremer, 2015)], bead assignment (i.e., the choice of the CG interaction parameters), and determination of the bonded parameters [as PyCGTOOL (Graham et al., 2017) or Swarm-CG (Empereur-mot et al., 2020)], allowing further coverage of chemical space. The creation of accurate databases and integration of automatic tools is currently one of the main bottlenecks hampering high-throughput screening with Martini.

## Virtual Screening: Martini Dynamic Docking

Virtual screening is the core of the drug design pipeline, and usually relies on docking algorithms. The use of Martini CG models will enable a new approach: dynamic docking with no *a priori* knowledge of the binding pocket in the target structure. The concept here is to sample protein–ligand interactions with CG MD simulations, which is around 300 to 1,000 times faster than atomistic MD (Souza et al., 2020). A practical example of such speed up can be given for propranolol binding to β2 adrenergic receptor, which has been simulated in atomistic (Dror et al., 2011) and coarse-grained (Souza et al., 2020) resolution. Atomistic simulations showed one binding event every 11.9 μs, which for a single simulation would take 84 days of computing time (using the 4 CPUs and 1 GPU in a computer/conditions described in the performance tests of Souza et al., 2020). The same system in CG simulations showed roughly the same number of binding events per μs (considering a normalization based in the different concentration of ligands), and would take 2 to 7 h of computing time, on the same hardware. We remark that a fair comparison between coarse-grained and atomistic simulation time is not trivial, since this should consider the different simulation conditions (e.g., ligand concentration) and parameters (Souza et al., 2020).

Multiple strategies are possible to accelerate sampling even more, with different computational costs and different levels of sophistication. Unbiased MD simulations could be applied in certain cases, to obtain not only binding poses but also estimates of binding affinities, as recently demonstrated for T4 lysozyme (Souza et al., 2020). However, for a general approach to virtual screening, faster methods are necessary. One possibility is to combine CG models with enhanced sampling techniques that do not depend on prior knowledge of the binding pathways. Examples are Gaussian accelerated molecular dynamics (GaMD) (Miao et al., 2015; Pang et al., 2017), and Hamiltonian Replica Exchange Molecular Dynamics (H-REMD) (Wang et al., 2013; Luitz and Zacharias, 2014). Computational performance can be straightforwardly increased by optimizing ligand concentration, to increase the probability of binding. The approach was already tested with atomistic simulations in a variety of systems (Dror et al., 2011; Shan et al., 2011; Decherchi et al., 2015; Schneider et al., 2016; Mondal et al., 2018). To avoid ligand aggregation, artificial repulsive interactions among ligands may be used (Shan et al., 2011). Similar strategies are also extensively used in so-called mixed-solvent (or co-solvent) approaches, where high concentrations of fragments are used to identify and stabilize cryptic pockets (Guvench and MacKerell, 2009; Bakan et al., 2012; Schmidt et al., 2019; Kuzmanic et al., 2020). Another idea is to only use isolated beads as probes representing chemical groups or fragments, to predict the chemical topology in pockets and generate pharmacophore models (Michelarakis et al., 2018; Michelarakis, 2019). The combination of CG models, enhanced sampling, and ligand/fragment concentration strategies will allow simulations of competitive binding assays.

An advantage of Martini dynamic docking approach is the improved representation of protein flexibility via Gō-like potentials (Poma et al., 2017; Souza et al., 2019). Although some docking strategies can also include protein flexibility (Amaro et al., 2018; Evangelista Falcon et al., 2019), they usually depend on prior sampling of the protein conformational space, followed by docking in a specific chosen pocket. In the strategy proposed here, no *a priori* selection of the binding pocket is needed. Both induced-fit and conformational-selection mechanisms are included in MD simulations, as recently demonstrated (Souza et al., 2020); however, accuracy will depend on the quality of the protein CG model.

Another major advantage is the possibility to include complex environments, such as multicomponent membranes, crowded protein solutions, or other relevant *in vivo-*like conditions, allowing more realistic predictions. Competition with the environment may be relevant for proper interpretation of ligand biological activity. For instance, lipid membrane composition may affect kinetic rates and (un)binding constants in GPCRs (Vauquelin, 2010; Sykes et al., 2014, 2019; Yuan et al., 2018) by altering ligand partitioning to the membrane where the target protein is located. Atomistic MD simulations of such complex systems are computationally very costly, while they are already within reach with Martini (Marrink et al., 2019).

Combining “standard” docking algorithms with Martini provides a computationally cheap alternative to all-atom docking. As recently demonstrated by HADDOCK (Honorato et al., 2019; Roel-Touris et al., 2019), docking with Martini can be one order of magnitude faster than atomistic docking. This would allow to routinely explore very large ligand datasets (Lyu et al., 2019) or even to use massive docking with grids covering the whole the protein, or exploring multiple proteins/conformations at the same time. However, common problems of docking approaches (mentioned above) still would be present; probably Martini MD approaches represent a better compromise between accuracy and computational performance.

## Lead Optimization: Backmapping and Coarse Graining in Chemical Space

**FIGURE 1:**
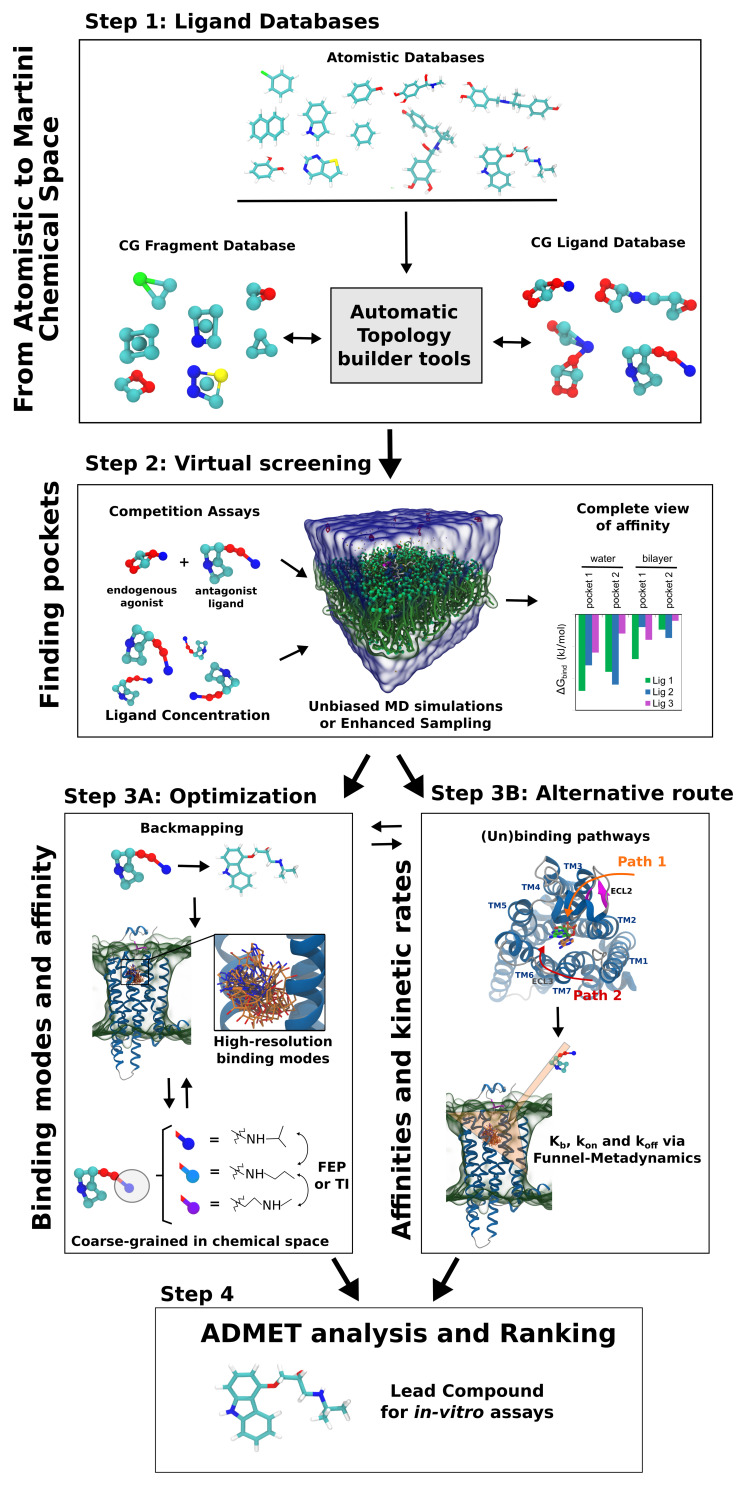
One of the possible pipelines for high-throughput dynamic docking based on Martini coarse-grained modeling. **(1)** The first step in the pipeline is the automatic conversion of input libraries of small compounds to Martini models. The library includes drug-like compounds and small-sized rigid molecules, useful for fragment-based drug discovery. **(2)** In the second step, thousands of parallel simulations are automatically set up, to sample small-molecule binding to pockets in the target protein. Competition *in silico* assays with endogenous ligands are possible in this step. Performance can be straightforwardly increased by optimizing the ligand concentration as well as by employing enhanced sampling techniques. At the end, automatic analysis and ranking of ligands is performed, to obtain estimates of binding affinity in relation to different pockets and environments (e.g., binding to protein in relation to water and/or bilayer). **(3A)** After defining the pocket and a set of candidates, the accuracy of the prediction can be improved in third step: backmapping to the atomistic models can be performed, providing high-resolution details of the binding modes. Additionally, free energy perturbation (FEP) or thermodynamic integration (TI) estimating the energetic cost of converting certain chemical groups into others can allow further optimization of the molecular structure. Here, coarse-graining in the chemical space is possible, as Martini CG moieties can represent more than one chemical fragment at the same time. **(3B)** An alternative or complementary third step, based on binding affinity and kinetics, is also considered here. Analysis of trajectories obtained in step 2 can help to identify the drug (un)binding pathways, which can be used in methods as Funnel-Metadynamics to provide lowest energy binding modes and dissociation rates k_off_ (drug residence time) states determining. **(4)** The combined analysis of steps II and III can be used for predictions of activity, which in combination with ADMET predictions leads to the final rankings and selection of the lead compounds for *in vitro* assays. Part of the figure is adapted from Souza et al. (2020).

Accurate predictions of ligand binding poses and affinities are key aspects for lead optimization (Jorgensen, 2009; Vivo et al., 2016). In atomistic pipelines, MD simulations can be used as a post-processing tool to validate and/or refine the binding poses from docking (Vivo et al., 2016). After this first check, more rigorous estimates of ligand binding affinities can be achieved by free energy perturbation (FEP) or thermodynamics integration (TI) (Jorgensen and Thomas, 2008; Jorgensen, 2009) – methods based on conversion of one ligand to another, allowing to add or replace substituents, in order to optimize ligand-protein interactions. In a Martini drug design pipeline (step 3A of Figure 1), one could simply convert the CG representation to all-atom (“backmapping” procedure) to verify and refine the CG docking poses. Currently, the most reliable approach for backmapping is the geometric projection implemented in *Backward* (Wassenaar et al., 2014). The main disadvantage is the need for mapping files for each ligand. After obtaining the atomistic structures, any MD-based simulations can be straightforwardly used. Careful equilibration is necessary to allow relaxation of the system, in particular, the water molecules may need to fill small cavities in pockets not accessible to CG water. One possibility is to model buried water molecules or ions using smaller beads, as previously showcased (Souza et al., 2020). Such difficulties are also common in standard docking approaches, as they usually do not include water molecules.

An alternative possibility for lead optimization in Martini would be to reverse the order of the steps, performing first a preliminary set of FEP/TI calculations at the Martini CG level. Such approach would allow to explore a broader portion of the chemical space. On top of the default computational efficiency of CG models, additional speed up could be obtained. First, given the smoother potential surface, the replacement of one bead for another (representing different chemical groups) could be performed in less FEP/TI windows. Additionally, as Martini CG beads generally represent more than one chemical fragment (Menichetti et al., 2019; Bereau, 2020), the exploration of chemical space increases computational efficiency by an additional factor 10^3^–10^4^ (Menichetti et al., 2019; Bereau, 2020) thanks to the reduction in the size of chemical space. Each bead of the CG model can be transformed into different chemical groups, for instance by using different mapping files for each bead in the Backward code (Wassenaar et al., 2014). With this alternative lead optimization approach, backmapping would be performed as the last step, to increase accuracy of the predictions.

## Alternative Route: Ligand Binding Pathways, Binding Affinities, and Kinetic Rates

Drug discovery is historically focused on the elucidation and optimization of the ligand binding mode and binding affinity. However, *in vivo* drug activity is quantitatively correlated to the drug residence time – i.e., dissociation constant rate k_off_ – more than binding affinity K_b_ (Copeland et al., 2006). The idea of integrating kinetic data in drug screening has been around since the beginning of 2000s (Limongelli, 2020; Nunes-Alves et al., 2020). However, ligand binding kinetics is determined by rare events, crossing ephemeral, high-energy states, elusive to both experiments, and computations (Copeland, 2016). The recent proof of concept with Martini 3 (Souza et al., 2020) opens the possibility of including information on ligand binding pathways in drug design pipelines (step 3B of Figure 1). The data coming from unbiased CG MD simulations should be integrated in a rigorous theoretical framework. One possibility would be to use Markov state models (Husic and Pande, 2018) based on Martini dynamic docking screening (step 2 of in Figure 1). The method has proven useful in atomistic ligand binding simulation studies (Buch et al., 2011) but it shows difficulties in defining the macrostates of the process, the choice of lag-time, and the sampling necessary to ensure statistical significance. An attractive strategy is to combine CG MD with Funnel-Metadynamics (Limongelli et al., 2013; Raniolo and Limongelli, 2020) that has emerged as a powerful method to reproduce binding mechanisms in ligand/protein and ligand/DNA complexes, identify crystallographic binding modes and predict binding free energies (Troussicot et al., 2015; Comitani et al., 2016; Moraca et al., 2017; Saleh et al., 2017; Yuan et al., 2018; D’Annessa et al., 2019). During FM simulations, the whole drug binding mechanism is reproduced, from the fully solvated state to the final binding mode, allowing to disclose important aspects of the binding process such as (i) the presence of alternative binding modes; (ii) the role of the solvent; and (iii) the kinetically relevant states (Tiwary et al., 2015; Brotzakis et al., 2019; Raniolo and Limongelli, 2020). CG-FM allows quantitative predictions of k_off_ and K_b_, ligand binding modes, and rate determining steps (Figure 1). This advance will represent a paradigm shift in drug design, as medicinal chemists would optimize the structure of drug candidates not only based on the static representation of the ligand binding mode, but also on the structures of kinetically relevant states. We point out that the reduction of friction from the missing atomistic degrees of freedom speeds up CG dynamics and affects kinetic estimates. However, estimating trends may be useful enough for ligand screening, while realistic kinetics rates might be recovered from estimates of the friction reduction (Español and Zúñiga, 2011).

## Further Considerations and Discussion

We described a new vision of high-throughput drug screening based on Martini CG models. Although most of the recent efforts in new drug design approaches focused on artificial intelligence (AI), the development of new methods covering gaps in standard approaches is equally important. Machine learning and other AI approaches have great advantages when tackling problems with enough experimental data to be used as training dataset. In situations where this is not the case, physics-based approaches (such as CG molecular dynamics) can perform better. In particular, structural databases of transmembrane proteins are still limited. The same is also true for databases that include dynamic information, which can be important to elucidate hidden allosteric pockets, to properly model fit-induced ligand binding process or to determine ligand association/dissociation pathways. More than complementary, AI and physics-based approaches can be combined, with CG MD simulations being used for the training of AI models or for the further refinement of AI predictions.

The proposed Martini drug design workflow (Figure 1) could be applied in full, or specific modules could be adapted in more traditional virtual screening campaigns. Screening of drugs based on ligand binding pathways and dissociation rates is currently out of reach for all-atom descriptions, due to the prohibitively high computational cost. Flexible proteins in complex environments are also too costly for all-atom docking approaches. Martini greatly reduces the computational costs of MD, while offering reasonable accuracy and structural detail. Accuracy will be further improved with the implementation of polarizable models (Yesylevskyy et al., 2010; de Jong et al., 2013; Michalowsky et al., 2017, 2018; Khan et al., 2020). Additionally, protonation state changes and pH effects can be included with Titratable Martini approaches (Grünewald et al., 2020). Also within reach is the design of epitopes and nucleic acids, useful for rational vaccine development (Kulp and Schief, 2013; Hodgson, 2020; Norman et al., 2020). In this context, even CG MD simulations may be overly expensive, as the approach demands scanning of protein-protein and protein-nucleic acid interfaces. Here, combination with standard docking is already a reality, as recently implemented in HADDOCK (Honorato et al., 2019; Roel-Touris et al., 2019). Overall, we believe dynamic docking with CG models has great innovation potential, both in academic and private sectors, and we hope this Perspective will contribute to motivate the modeling community to expand the efforts in this area.

## Data Availability Statement

The original contributions presented in the study are included in the article/supplementary material, further inquiries can be directed to the corresponding authors.

## Author Contributions

PCTS wrote the first draft of the manuscript and prepared the figure. All authors contributed to the conception of the perspective article, manuscript revision, read, and approved the submitted version.

## Conflict of Interest

The authors declare that the research was conducted in the absence of any commercial or financial relationships that could be construed as a potential conflict of interest.

## Footnotes

1 mad.ibcp.fr
